# Avoidance of external fixation pin induced rotational stiffness in the forearm; a cadaver study of soft tissue displacement relative to the varying position of radius and ulna fixation

**DOI:** 10.1051/sicotj/2015005

**Published:** 2015-05-29

**Authors:** Pavel Nikolaevich Kulesh, Matt D.A. Fletcher, Leonid N. Solomin

**Affiliations:** 1 Vreden Russian Research Institute of Traumatology and Orthopedics 8 Baykova Str. St. Petersburg 195427 Russia; 2 Dawson Creek and District Hospital 11100-13th Street Dawson Creek BC V1G 3W8 Canada; 3 St. Petersburg State University Universitetskaya 7-9 St. Petersburg 199034 Russia

**Keywords:** Rotation stiffness, Forearm rotation, Transfixion pin-induced stiffness, Transosseus osteosynthesis, External fixation

## Abstract

*Introduction*: Stiffness of forearm rotation secondary to transfixion pin sites is a frequent complication of external fixation. Conventional surgical atlases do not consider the effect of rotation on skin displacement and thus do not provide a comprehensive answer. We asked: (1) in what locations in the forearm is soft tissue displacement relative to the ulna and radius least during rotation; (2) in what positions are major neurovascular structures absent; and (3) what maximal range of rotation can be expected in forearm external fixation.

*Methods*: Thirty-four matched cadaver arms were used to assess displacement of soft tissues at 10°, 30° and 70° of pronation and supination in relation to a testing frame. The results of these were correlated with positions in which neurovascular structures were absent and deemed insertional “Reference Positions (RP)”.

*Results*: Expected range of rotation in diaphyseal fractures of different levels of both forearm bones was found with RP for the ulna occurring along the length of the forearm. Reference positions for the radius which provide full forearm rotation are situated only in the distal third; positions which provide partial rotation are located in the proximal and middle third.

*Discussion*: Full range of rotation may be maintained in the case of isolated external fixation of ulnar diaphyseal fractures. In isolated external fixation of the radius a reduced range of forearm rotation may be expected.

## Introduction

Treatment of isolated or combined forearm injury and deformity is typically performed with rigid internal fixation with temporary external fixation frequently used in the initial management of severe soft tissue trauma, however definitive external fixation of the forearm is performed much less commonly for a number of reasons; the high frequency of transfixion pin-induced (and in particular, rotational) stiffness being one of the more important [3, 4, 6, 11–14, 16–18]. This stiffness frequently occurs due to the fixation of soft tissues to fine wires and half-pins. Loss of pronation and supination in the forearm carries significant functional deficits and may jeopardize otherwise excellent clinical results [8, 10]. Most atlases of insertion of external fixation pins and wires use “safe corridors” and “safe positions” without considering soft tissue displacement, thus neglecting to consider the effect of pin site placement upon functional range of motion during and subsequent to treatment [2, 5, 6, 9, 13].

External fixation has proved itself to be invaluable in the management of complex pathology of other parts of the appendicular skeleton [10, 15], to date there has been little investigation of the potential in the forearm. Internal fixation of the forearm is not free of complications, and in particular, proximally, surgical approach and risk to neurological structures can be significant [3, 7, 14].

Diaphyseal fractures of the forearm treated functionally with external fixation are unique in that they are extra articular and subject to an unusual range of motion with subsequent soft tissue displacement. The use of individual or isolated external fixation of the radius and/or ulna has not to date prevented pin-induced rotation stiffness.

The aim of this study was to discover reference positions (RP) for the insertion of external fixation elements, which fulfilled two conditions, namely the absence of significant neurovascular structures, and minimal displacement of the soft tissues during forearm rotation, and hence predicting the achievable range of forearm rotation for each RP.

## Methods

Using 34 pairs of matched cadaveric upper limbs without musculoskeletal pathology, the displacement of skin, fascia and muscles relative to ulna and radius at 70° of pronation and 70° of supination was studied. The age of the cadaveric material used was elected to be standardized from 20 to 40 years as increased soft tissue laxity in the older adult and flexibility in the young may permit unpredictable increased soft tissue deflection. Forearm circumference could not be standardized due to the limited cadaveric material available. Preliminary study data demonstrated that soft tissue displacement around the radius was great and precluded full rotation of the forearm. The study was therefore modified to additionally evaluate skin displacement at 10° and 30° of pronation and of supination, respectively.

Soft tissue displacement relative to the ulna was studied on one forearm, and relative to radius on the other matched forearm. The rationale for the separate forearm studies was to exclude artefactual false skin, fascial and muscular movement due to the releasing effect of incisions for frame construct application. There was equal distribution of left and right radii and ulnae within the study. The initial forearm position was in neutral, and the elbow was fixed in extension as in flexion forearm tissue tension is frequently diminished and thus would result in an unachievably high expected range of motion when the elbow was held in extension.

**Figure 1.**
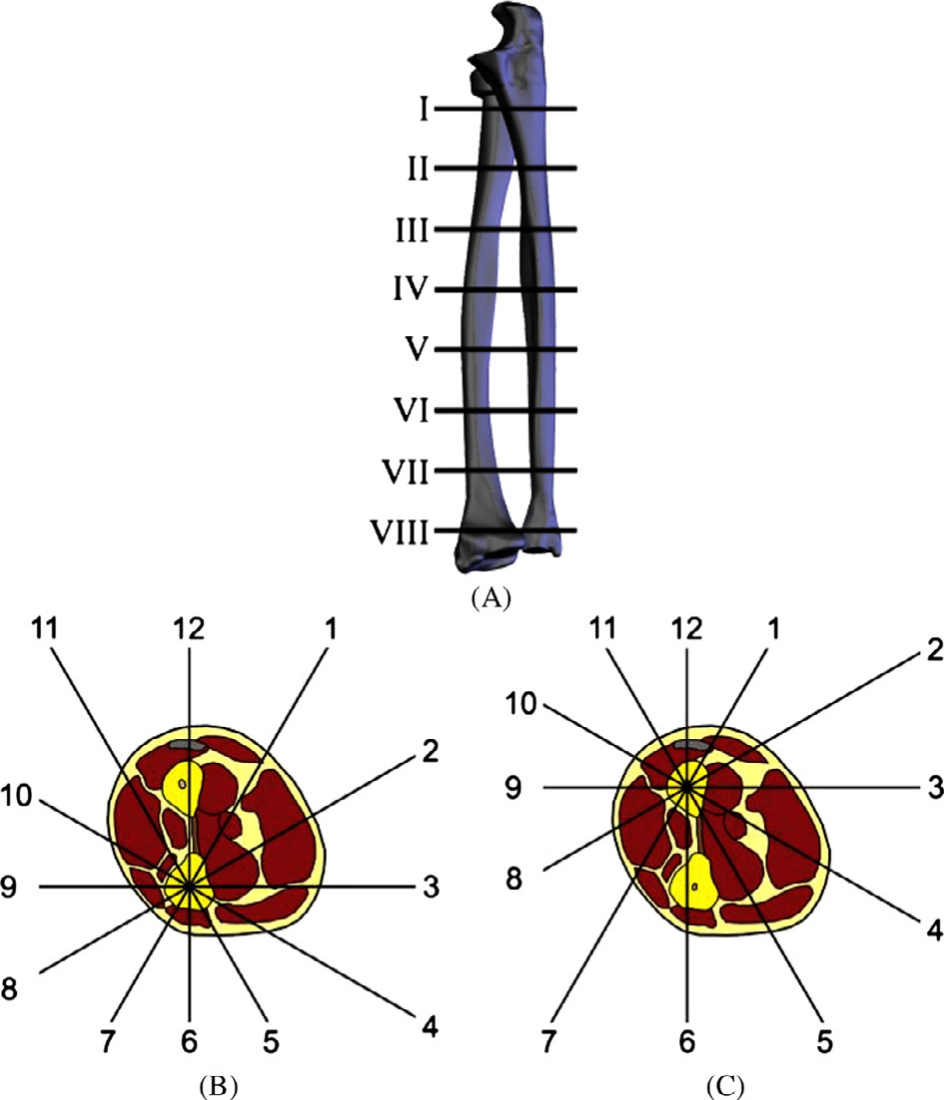
(A) Diagram of forearm division into eight principal levels according to MUDEF system of coordinates. (B) Forearm, level IV: designation of positions relative to the ulnar bone. (C) Forearm, level IV: designation of positions relative to the radial bone.

The system of coordinates of the Method of Unified Designation of External Fixation (MUDEF) was used [1, 15, 16]. According to this system the forearm is divided into eight equidistant levels (Figure 1A). Level I corresponds to the neck of the radius. Level VIII corresponds to the distal metaepiphysis of the radius. Each level has 12 positions (Figures 1B and 1C). Position 3 is situated along the ventral surface of the segment, 9 – along the dorsal, 6 – along the ulnar, 12 – along the radial surface of the segment. Therefore, soft tissue displacement in 96 positions for each bone was measured.

**Figure 2.**
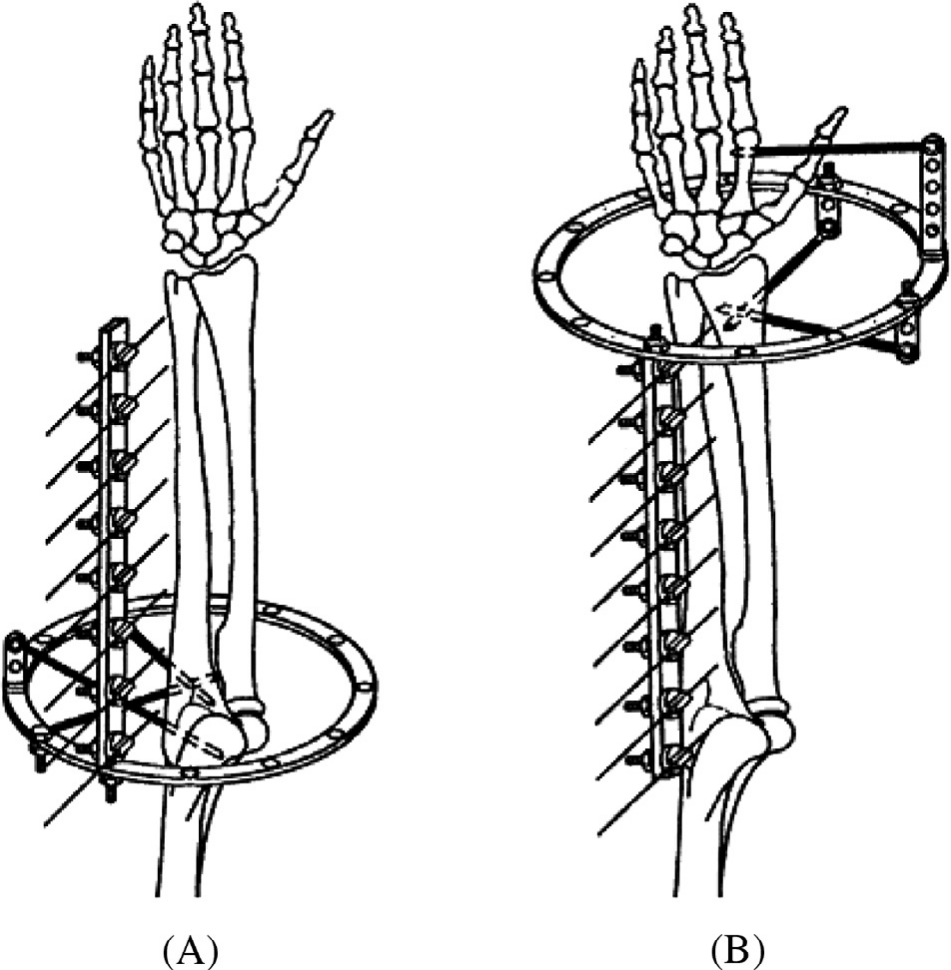
(A) Schematic illustration of the device used to study soft tissue displacement in rotation relative to the ulnar bone. (B) Schematic illustration of the device used to study soft tissue displacement in rotation relative to the radial bone.

A specially constructed device (Figure 2A) was used to study soft tissue displacement relative to the ulna. Two half-pins were inserted into the posterior aspect of the proximal metaphysis of the ulna (position 6 at levels I and II). A third half-pin was inserted through olecranon into the metaphysis of the humeral bone to stabilize the elbow in extension. The ring support was applied perpendicular to the axis of the ulna, with the ulna central. Wire guide supports (wire fixation bolt with K-wire inserted) were rigidly attached to the device. Guide supports were sequentially placed in the projections of all eight levels.

A similar device was used to study soft tissue displacement relative to the radius (Figure 2B). Two half-pins were inserted into the radial aspect of the distal metaepiphysis of the radius (position 12 at levels VII and VIII). An additional half-pin was placed into the second metacarpal to eliminate wrist motion. The ring support was applied perpendicular to the axis of the radius, and radius was centralized. A wire guide support bar was attached to the ring.

**Figure 3.**
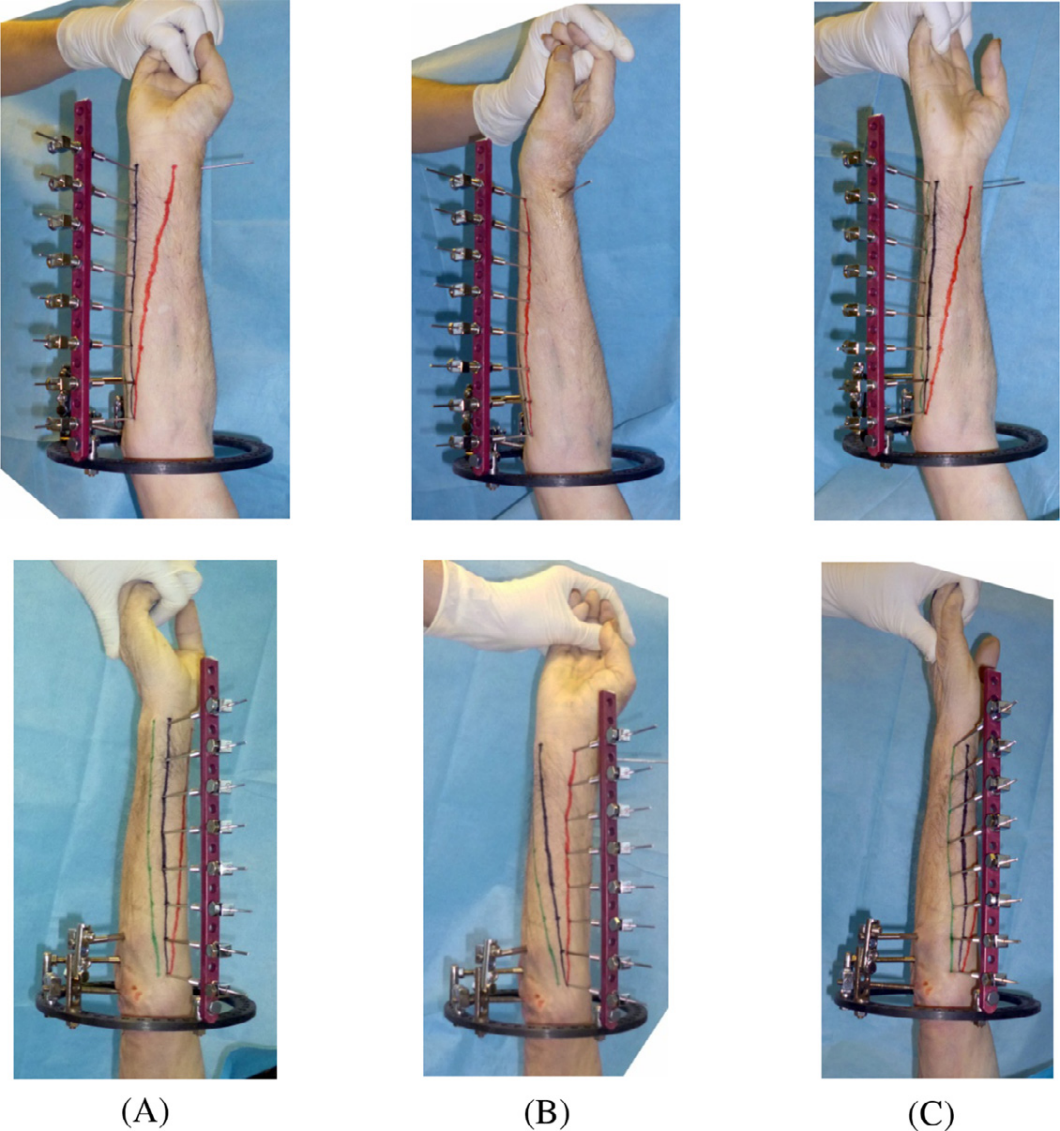
(A) Initial position relative to the ulna in the projection of position 5 (black line). (B) Pronation of forearm up to 70° (red line). (C) Supination of forearm up to 70° (green line).

The forearm was placed into neutral position between supination and pronation. The wire guide support bar was assembled and affixed such that each guide support was in the projection of position 1 at each level. Ink was applied at the ends of guide wires; all the wires were advanced until they contacted the skin; the resulting marks were connected (line 1). The wires were retracted; the forearm was rotated and stabilized. Then the guide wires were advanced a second time until they contacted skin; a second line was obtained (line 2). We measured the distance between the dots of the first and second lines at each level and derived the value of *skin* displacement in rotation for position 5 at each of eight levels. Using the aforementioned protocol, the sequential study of skin displacement in each position at each level was performed. Figure 3 demonstrates, for example, the study of skin displacement relative to the ulna in the axis of position 5 for all eight levels.

Subsequently, the skin later was removed and fascial displacement was studied using the same protocol. Finally, the fascia was removed, and muscular displacement was evaluated.

Descriptive statistics were run on all the variables, means and standard deviations, and percentiles. We used the Student’s *t*-test with a 95% confidence interval for significance testing (*t*_0.95, 34_ = 1.6909 (*α* = 0.2, *k* = 4)). Positions with soft tissue displacement of less than 10 mm were chosen at each level. If blood vessels and nerves were absent in the projection of the position with minimal soft tissue displacement, this position was deemed a “Reference Position” for wires and half-pins insertion [1].

## Results

Table 1 displays the values of each soft tissue displacement relative to the ulna at 70° of pronation and 70° of supination. Of 96 possible positions for the ulna there are 33 (34%) with minimal (up to 10 mm) soft tissue displacement and 31 (32%) which meet criteria for a “Reference Position” (Table 2).

**Table 1.** Displacement of skin/fascia/muscles [mm] relative to the ulna at 70° of pronation and 70° of supination.

Position	Level							
	I	II	III	IV	V	VI	VII	VIII
1	*9/9/11*	*17/10/16*	*19/21/18*	*37/18/24*	*42/30/24*	*48/31/33*	*51/50/41*	*58/48/38*
	*10/7/10*	*11/13/20*	*17/16/17*	*20/24/23*	*22/26/31*	*34/36/29*	*36/37/38*	*52/44/40*
2	*8/11/7*	*14/12/11*	*15/19/15*	*30/21/20*	34/27/34	*44/29/40*	*46/29/39*	*58/40/33*
	*5/5/15*	*8/10/13*	*16/14/22*	*21/20/28*	21/25/27	*32/33/28*	*32/39/32*	*52/25/38*
3	***8/7/0***	***9/7/7***	12/15/18	*23/20/17*	*26/18/26*	*33/24/28*	*43/24/36*	*52/39/32*
	***3/0/0***	***3/1/0***	6/5/4	*9/11/6*	*12/18/9*	*12/15/4*	*15/22/9*	*16/33/18*
4	**6/3/0**	**7/4/0**	**9/7/3**	18/8/15	17/10/20	19/13/6	33/20/5	*38/22/4*
	**2/0/0**	**2/0/0**	**5/2/0**	6/4/5	9/8/3	10/9/0	18/13/0	*19/15/4*
5	**2/1/0**	**2/4/0**	**3/3/0**	**9/6/7**	**10/7/4**	**10/5/2**	18/12/0	15/19/0
	**0/0/0**	**0/0/0**	**3/1/0**	**6/4/3**	**6/5/0**	**9/6/0**	13/9/0	15/20/0
6	**1/0/0**	**1/0/0**	**4/1/0**	**5/1/0**	**5/3/0**	**7/3/0**	**9/6/0**	**10/10/0**
	**0/0/0**	**0/0/0**	**4/0/0**	**5/3/0**	**7/5/0**	**9/5/0**	**10/6/0**	**9/10/0**
7	**2/0/0**	**3/0/0**	**3/3/0**	**3/2/0**	**6/4/3**	6/4/0	**10/9/0**	17/12/0
	**0/0/0**	**0/0/0**	**3/1/0**	**8/4/0**	**6/5/1**	13/6/0	**9/8/0**	16/10/0
8	**2/0/0**	**4/1/0**	**6/1/0**	**5/4/0**	5/4/1	7/5/0	13/4/0	34/14/2
	**0/0/0**	**3/0/0**	**6/2/5**	**8/7/6**	13/8/9	19/13/3	13/7/3	13/12/3
9	**4/1/0**	**8/3/0**	10/4/2	9/8/2	7/4/2	12/8/1	12/11/3	37/15/5
	**2/0/0**	**5/0/0**	9/5/17	17/9/12	17/16/17	30/16/18	21/25/15	25/19/1
10	**9/3/2**	**9/2/3**	17/7/11	15/10/5	15/128/	19/15/4	21/17/11	42/14/14
	**1/0/3**	**8/2/7**	15/14/24	23/20/33	34/22/27	36/30/44	38/50/45	41/32/19
11	11/7/3	16/8/9	23/12/14	27/22/20	32/24/21	30/22/20	41/24/34	50/24/25
	3/7/5	13/8/15	20/22/25	24/23/28	21/38/40	38/43/49	44/59/49	43/34/30
12	*10/10/7*	*21/7/10*	21/13/16	34/19/21	39/26/28	39/33/28	45/37/47	55/44/42
	*5/3/10*	*9/7/15*	17/19/18	16/19/29	20/30/35	31/45/36	39/48/41	42/39/55

Here and further: italic type marks the forbidden positions, bold type marks the positions with minimal soft tissue displacement, bold and italic type marks the forbidden positions with minimal soft tissue displacement.

**Table 2.** Groups of positions for external fixation insertion.

Level	Positions
I	4–10
II	4–10
III	4–8
IV	5–8
V	5–7
VI	5, 6
VII	6, 7
VIII	6

Table 3 represents the values of soft tissue displacement relative to the radius at 70° of pronation and at 70° of supination, respectively. It was found that in rotation the skin moved a greater amount than fascia and muscles. Thus, at 10° and 30° of pronation and 10° and 30° of supination skin displacement only was studied.

**Table 3.** Displacement of skin/fascia/muscles [mm] relative to the radius at 70° of pronation and 70° of supination.

Position	Level							
	I	II	III	IV	V	VI	VII	VIII
1	*73/54/57*	*60/36/39*	50/26/33	40/22/38	28/11/20	28/11/23	16/6/8	**9/1/4**
	*61/50/45*	*53/43/48*	43/30/29	37/21/29	31/19/23	25/8/15	16/4/3	**10/1/4**
2	*69/59/54*	*55/38/31*	*46/40/32*	*35/22/32*	*26/19/20*	*24/13/22*	*18/6/11*	***10/6/9***
	*65/45/42*	*58/45/52*	*46/24/30*	*42/23/35*	*32/19/24*	*27/12/18*	*18/11/7*	***10/6/8***
3	*72/56/46*	*52/48/31*	*43/34/32*	*32/31/30*	*25/24/21*	*20/14/18*	*17/5/13*	*16/8/13*
	*63/60/52*	*60/44/57*	*45/33/40*	*47/28/40*	*33/20/29*	*30/21/22*	*19/15/12*	*15/7/13*
4	*68/46/48*	*54/36/28*	*40/37/33*	*35/27/30*	*27/20/22*	*26/14/16*	*18/9/16*	*20/4/6*
	*68/55/64*	*70/59/58*	*55/44/48*	*47/37/42*	*37/34/30*	*31/21/26*	*20/15/14*	*18/4/7*
5	55/46/48	56/34/30	39/28/23	*40/25/26*	*29/19/25*	*22/14/17*	*19/8/15*	*10/5/3*
	64/55/47	60/50/51	65/47/59	*54/42/46*	*37/34/31*	*28/26/27*	*25/18/20*	*14/5/3*
6	50/45/45	50/28/29	42/25/20	37/21/21	30/16/28	26/12/20	21/7/12	17/1/0
	60/48/43	61/54/49	61/44/43	56/28/28	38/30/26	26/19/21	21/17/16	10/1/0
7	56/44/44	46/32/26	37/24/23	50/17/24	41/16/35	21/10/18	16/5/12	18/9/2
	52/49/48	59/52/52	55/40/36	47/35/35	37/25/29	29/23/25	22/16/18	10/9/2
8	74/41/48	40/22/27	40/22/24	56/23/32	49/10/35	28/7/11	21/7/14	24/1/0
	50/43/46	50/47/48	41/31/35	44/34/37	40/22/30	27/13/20	20/9/17	7/1/0
9	80/39/49	76/30/47	65/21/23	53/22/38	46/11/33	35/3/8	21/4/16	12/1/0
	40/32/33	40/35/37	34/30/30	35/30/28	27/23/22	16/14/12	15/7/12	7/1/0
10	88/43/54	55/30/38	57/24/26	48/19/37	40/12/30	28/5/10	23/3/8	15/0/0
	45/39/39	48/42/46	39/34/32	33/28/29	26/15/18	14/8/9	13/7/3	10/0/0
11	86/44/61	*70/31/42*	60/27/28	46/19/36	35/14/26	28/4/15	17/2/5	13/0/0
	56/50/46	*53/46/47*	45/34/37	35/27/28	28/21/23	15/9/10	16/5/3	8/0/0
12	80/55/58	*70/29/41*	56/23/27	45/20/43	32/10/22	35/6/20	15/4/4	**10/0/3**
	58/35/49	*50/35/45*	40/35/33	37/26/29	30/23/27	28/9/14	18/2/3	**10/0/2**

Table 4 demonstrates the values of skin displacement relative to the radius at 10° and 30° of pronation, and at 10° and 30° of supination, respectively. Of 96 positions for the radius there are three (3%) with minimal soft tissue displacement. Only two (2%) positions meet criteria for reference position. These are situated at level VIII in positions 1 and 12.

**Table 4.** Skin displacement [mm] relative to the radius at 10°/30°/70° of pronation and 10°/30°/70° of supination.

Position	Level							
	I	II	III	IV	V	VI	VII	VIII
1	*22/43/73*	*18/36/60*	14/27/50	**8**/21/40	**7**/15/28	**8**/19/28	**5/10**/16	**3/6/9**
	*13/33/61*	*13/31/53*	14/26/43	**10**/20/32	**9**/18/31	**7**/14/25	**4/9**/16	**3/7/10**
2	*17/36/69*	*16/30/55*	*15/25/46*	***10*** */20/35*	***6*** */13/26*	***6*** */14/24*	***4/9*** */18*	***2/5/8***
	*12/33/65*	*12/30/58*	*15/27/46*	*11/21/37*	***10*** */19/32*	***8*** */16/27*	***5/10*** */18*	***4/7/10***
3	*18/38/72*	*15/27/52*	*15/24/43*	*11/20/32*	***6*** */13/25*	***4/9*** */20*	***3/7*** */17*	***2/5/10***
	*12/30/63*	*12/29/60*	*12/24/45*	*11/22/39*	*11/20/33*	***9*** */18/30*	**5** */11/19*	***4/9*** */15*
4	*17/36/68*	*16/29/54*	*13/21/40*	***10*** */20/35*	***7*** */15/27*	***8*** */16/26*	***4/9*** */18*	***4/10*** */20*
	*13/32/68*	*15/31/70*	*17/30/55*	*13/24/42*	*11/21/37*	***9*** */20/31*	**7** */12/20*	***5*** */13/18*
5	15/32/55	17/32/56	14/22/39	*16/29/40*	***9*** */18/29*	***7*** */14/22*	***5*** */12/19*	***2/5/10***
	16/33/64	18/33/60	21/37/65	*15/29/49*	*12/22/37*	***9*** */19/28*	***10*** */19/25*	***4/10*** */14*
6	15/30/50	17/30/50	17/28/42	13/25/37	11/20/30	**8**/16/26	**6**/14/21	**5/10**/17
	18/35/60	20/35/61	20/35/61	17/30/51	13/23/38	**8**/17/26	**4/10**/21	**3/8/10**
7	17/34/56	17/28/46	15/25/37	17/40/50	14/30/41	**7**/13/21	**4**/11/16	**7**/12/19
	16/30/52	19/33/59	17/30/55	13/24/42	12/22/37	**9**/19/29	**4/9**/22	**3/7/10**
8	20/50/74	13/24/40	17/28/40	20/46/56	15/35/49	**8**/19/28	**6**/14/21	11/16/23
	14/35/50	14/26/50	12/23/41	13/24/39	13/24/40	**8**/18/27	**3/8**/20	**2/5/7**
9	31/62/81	25/60/76	23/50/65	18/42/53	14/35/46	**9**/25/35	**6**/14/21	**4/9**/12
	12/20/40	**10**/20/40	**8**/18/34	**7**/17/30	**5**/13/27	**4/10**/16	**3/7**/15	**2/5/7**
10	33/67/88	18/30/55	21/45/57	12/32/48	11/28/40	**8**/19/28	**7**/16/23	**7**/12/15
	12/25/45	14/27/48	11/23/39	**8**/17/28	**6**/14/26	**3/9**/14	**3/8**/13	**3/8/10**
11	32/64/86	*20/40/70*	22/47/60	**10**/28/46	**9**/21/35	**8**/19/28	**4/10**/17	**6**/11/13
	13/30/56	*16/32/53*	14/27/45	**8**/18/30	**7**/15/28	**4/9**/15	**4/9**/16	**2/6/8**
12	28/58/80	*21/43/67*	14/31/56	**10**/25/45	**8**/17/32	**9**/25/35	**5**/11/15	**4/8/10**
	14/32/58	*15/30/50*	13/24/40	**10**/20/32	**9**/17/30	**8**/16/28	**4/10**/18	**3/7/10**

Reference positions with the ability to achieve 10° of pronation are located at levels from IV to VIII; 10° of supination – from II to VI; and for 10° of both pronation and supination from levels IV to VI. Reference positions with the ability to achieve 30° of pronation are located at levels VII and VIII; 30° of supination at levels VI and VII; and for 30° of both pronation and supination – at level VII (Table 5).

**Table 5.** Range of *predictable* rotation in external fixation of the radius* (maximal pronation, neutral, maximal supination).

	I	II	III	IV	V	VI	VII	VIII
1	–	–	–	**10/0/10**	**10/0/10**	10/0/10	**30/0/30**	**70/0/70**
8	–	–	–	–	–	10/0/10	10/0/30	0/0/70
9	–	**0/0/10** **	**0/0/10**	0/0/10	0/0/10	**10/0/30**	10/0/30	30/0/70
10	–	–	–	0/0/10	0/0/10	**10/0/30**	10/0/30	10/0/70
11	–	–	–	**10/0/10**	**10/0/10**	**10/0/30**	**30/0/30**	10/0/70
12	–	–	–	**10/0/10**	**10/0/10**	10/0/10	10/0/30	**70/0/70**

* Positions 5–7 were not taken into account. These positions cannot be used for external fixation: in rotation the elements of external fixation frame will come in contact with the ulna (Solomin).

** Bold type marks the positions with maximal range of rotation for each level.

## Discussion

Whilst the Ilizarov method has found strong support in the limb reconstruction community in the management of complex combined bone and soft tissue trauma, bone loss, deformity and limb length inequality, the application of the method to the forearm had been limited by functional considerations. The development of a protocol to maximize functional range of motion both during and after treatment would be of significant benefit as this has been one of the main detractors of its use to date. This study has demonstrated that there are precise RP which can be used to reliably predict either full or a reduced rotational range of motion, and zones of avoidance have been clearly demonstrated.

**Figure 4.**
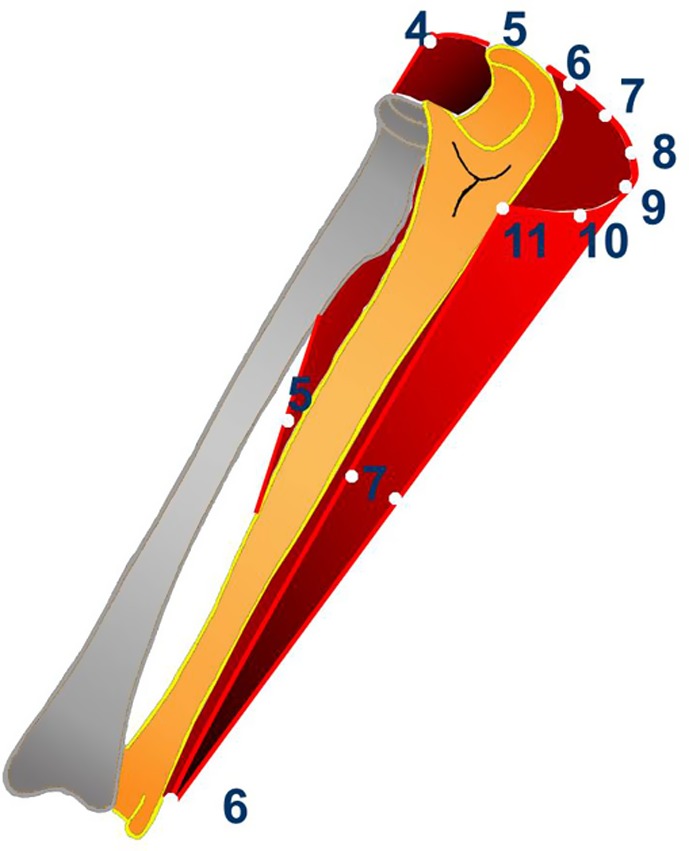
Reference positions for maximal range of motion in the ulna. Levels I–III positions 4–10. Levels IV–VI positions 5–7. Levels VII and VIII position 6.

Reference position for the *ulna* can be found at each of the eight levels. These are situated in triangular configuration along the dorsal aspect of the segment. Their number decreases distally: from seven RP at levels I and II to one RP at level VIII (Figure 4). Therefore, the number and distribution of RP allows application of external fixation to the ulna and the frame permits maintenance of full rotation during fixation. It is worth mentioning that at levels I and II there are RP which are diametrically opposed in positions 4 and 10. This means that either K-wire or half-pin fixation can be used. In all other cases only half-pins can be permitted.

**Figure 5.**
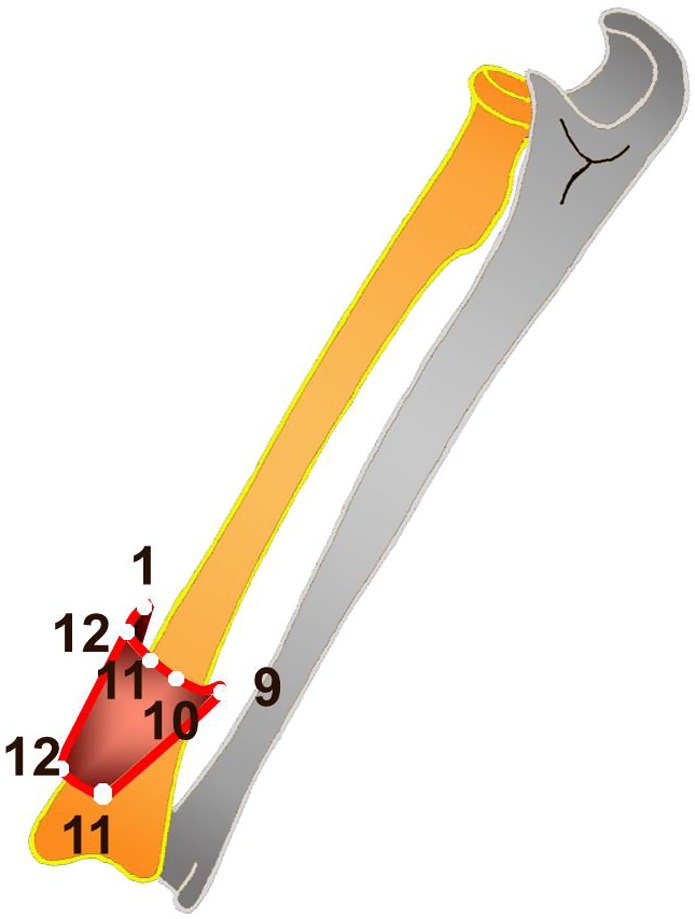
Reference positions for maximal range of motion in the radius. Level VII positions 9–12, 1. Level VIII, positions 11, 12, 1.

Reference position for the *radius* which permit full forearm rotation are located only at level VIII. Thus, it is impossible to maintain full range of rotation with external fixation of the radius. Also it is impossible to maintain 30° of pronation and 30° of supination (RP permitting this range are situated only at levels VII and VIII) (Figure 5).

Using the derived RPs, in external fixation of the middle third radial injuries the permitted range of rotation is 0/0/10 (the proximal half-pin should be inserted in position II, 9 or III, 9). In case of distal third injury permitted range is 10/0/10 (the proximal half pin should be inserted in positions IV, 1; IV, 11; IV, 12 or V, 1; V, 11; V, 12).

It was apparent that skin displacement was greater than fascial or muscular displacement during the course of the study, and this can be explained relative to the arc produced by increasing the radius of the degree of motion applied over the segment, and can be anticipated if the bone studied is located in the centre of the fixation device. Eccentric placement of the bone within a fixator will alter the radius and thus the degree of tissue displacement.

## Conclusion

This study demonstrates a major disparity between functional range of motion with external fixation of the forearm; with isolated stabilization of the ulna, functional range of motion can be planned, predicted and encouraged. Conversely, stabilization of the radius results in a very significant reduction of rotational range of motion that can be expected without major soft tissue displacement and development of symptoms. This has an important bearing on the present utility of external fixation of radial segment fractures.

Further research into forearm external fixation is necessary, with engineering of frame assemblies to increase the reduction characteristics and stabilization potential of frame management whilst permitting maximal range of motion by taking into consideration the reference positions this study has discovered.

## Conflict of interest

PNK, MDAF and LNS declare no relationships which might lead to a conflict of interest.

## Ethical review committee statement

Each author certifies that his or her institution has approved or waived approval for the human protocol for this investigation and that all investigations were conducted in conformity with the ethical principles of research and the Declaration of Helsinki.
